# The emerging role of auxins as bacterial signal molecules: Potential biotechnological applications

**DOI:** 10.1111/1751-7915.14235

**Published:** 2023-04-28

**Authors:** Tino Krell, José A. Gavira, Amalia Roca, Miguel A. Matilla

**Affiliations:** ^1^ Department of Biotechnology and Environmental Protection, Estación Experimental del Zaidín Consejo Superior de Investigaciones Científicas Granada Spain; ^2^ Laboratory of Crystallographic Studies IACT (CSIC‐UGR) Armilla Spain; ^3^ Department of Microbiology, Facultad de Farmacia Universidad de Granada Granada Spain

## Abstract

Microorganisms are exposed in their natural niches to a wide diversity of signal molecules. Specific detection of these signals results in alterations in microbial metabolism and physiology. Auxins like indole‐3‐acetic acid are key phytohormones that regulate plant growth and development. Nonetheless, auxin biosynthesis is not restricted to plants but is ubiquitous in all kingdoms of life. This wide phylogenetic distribution of auxins production, together with the diversity of regulated cellular processes, have made auxins key intra‐ and inter‐kingdom signal molecules in life modulating, for example microbial physiology, metabolism and virulence. Despite their increasing importance as global signal molecules, the mechanisms by which auxins perform their regulatory functions in microorganisms are largely unknown. In this article, we outline recent research that has advanced our knowledge of the mechanisms of bacterial auxin perception. We also highlight the potential applications of this research in aspects such as antibiotic production, biosensor design, plant microbiome engineering and antivirulence therapies.

## Graphical abstract

Considerable progress has been made in determining the mechanisms of auxins recognition by different bacterial sensor proteins. This knowledge has potential applicability, for example, for the identification of novel antibiotics, biosensor design, metabolism modulation, plant microbiome engineering and antivirulence therapy.

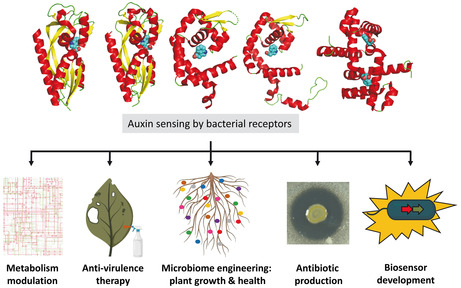

Microbes are subjected in their ecological niches and natural hosts to a wide diversity of physical, chemical and biological signals (Matilla et al., 2022; Webster et al., 2022). The perception of these signals, and the generation of an optimal response, is essential for microbial survival in highly competitive and challenging environments. Signal perception is carried out by a wide spectrum of signal transduction systems (Gumerov et al., 2020; Matilla et al., 2022) and genes involved in these regulatory cascades can account for more than 10% of the total genome of a bacterium (Galperin, 2018; Gumerov et al., 2020). Notably, environmental bacteria contain a particularly high number of signal transduction systems (Alexandre et al., 2004; Galperin, 2018; Gumerov et al., 2020), most probably reflecting the need for these microorganisms to adapt more efficiently to a greater number of environmental signals.

There do not appear to be limits to the diversity of signal molecules that include, for example, organic acids, amino acids, polyamines, phenolic compounds, aromatic acids, sugars, inorganic ions or different gases (Berlanga‐Clavero et al., 2020; Matilla et al., 2022). Thus, thousands of chemical signals are present, for example, in rhizospheric environments and in gastrointestinal tracts which play pivotal roles in plant‐microorganism interactions as well as in assembly and function of the gut microbiome, respectively (Berlanga‐Clavero et al., 2020; Vives‐Peris et al., 2020; Zheng et al., 2022). Among these signals, there is a considerably large body of experimental evidences on the role of indole as an inter‐ and intra‐kingdom signal molecule that modulates diverse metabolic and physiological functions in bacteria, including resistance to a broad range of stresses, biofilm formation, persister cells formation and chemotaxis (Han et al., 2021; Kumar et al., 2021; Lee et al., 2015; Song & Wood, 2022; Wood, 2022; Yang et al., 2020; Zarkan et al., 2020). In this regard, another indolic compound, the auxin indole‐3‐acetic acid (IAA), is emerging as a global signal molecule. IAA is a key phytohormone that regulates plant growth and development, among other plant processes (Gallei et al., 2020; Zhao, 2018). Nonetheless, auxin biosynthesis is not restricted to plants but is widely distributed in all kingdoms of life. Extraordinarily, the ability to synthesize IAA is widely distributed in bacteria that establish interactions with plants (Duca & Glick, 2020) and microbial IAA plays an important role during the interaction between microbes and their plant hosts (Duca et al., 2014; Duca & Glick, 2020; Kunkel & Johnson, 2021). Remarkably, a growing body of data supports the key role for IAA as an important signal molecule in bacteria that regulates, among other processes, stress resistance, biosynthesis of antibiotics and virulence factors, nutrient transport and bacterial catabolism (Cassan et al., 2021; Duca et al., 2014; Duca & Glick, 2020; Kunkel & Johnson, 2021; Laird et al., 2020). For example, recent research on the plant pathogenic bacterium *Pseudomonas syringae* revealed that IAA acts as a signal that modulates bacterial virulence in vitro and *in planta* (Djami‐Tchatchou et al., 2022). However, despite the pathways by which IAA carries out these regulatory functions in bacteria are largely unknown, recent research highlighted below has contributed significantly to deciphering the corresponding molecular mechanisms.

IAA is metabolized by a variety of microorganisms, including many plant and soil bacterial isolates that use this auxin as a nutritional and energy source (Laird et al., 2020). Typically, this IAA degradative capacity is highly regulated and the mechanisms by which different transcriptional regulators carry out their function have recently been established by Conway et al. (2022), using a multidisciplinary approach. The authors found that different MarR‐type transcriptional regulators associated with IAA degradation loci bind IAA and additional auxins with high affinity. MarR regulators from auxin catabolic operons were phylogenetically classified into three different groups, named IacR, IadR and IadR2. Only regulators from the IacR and IadR groups were found to bind different auxins (Conway et al., 2022). Crystal structures were solved for five different IacR and IadR regulators in the presence and absence of IAA and other auxins (Figure 1), which resulted in the identification of key residues for the coordination of these signal molecules. It was found that these MarR regulators act as repressors of the IAA catabolic operons, with IAA playing an essential role in the transcriptional de‐repression (Conway et al., 2022). The authors also conducted *in planta* signal interference assays to show that the root growth inhibition caused by high IAA levels is alleviated by IAA degrading root‐associated bacteria from six different genera (Conway et al., 2022) – highlighting the important ecological role of IAA catabolism in plant environments, as well as the enormous potential of engineering plant microbiomes to promote plant growth and health.

**FIGURE 1:**
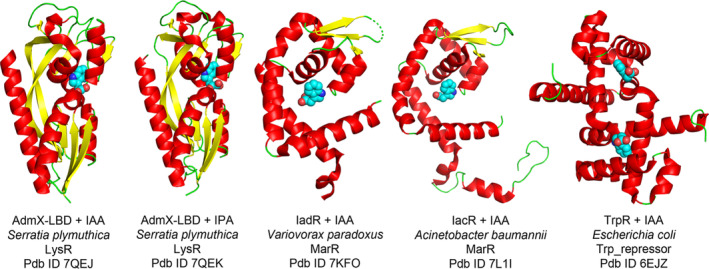
Three‐dimensional structures of bacterial auxin sensor proteins or sensor domains. Bound auxins are shown in the spheres mode. The bacterial species, protein families and protein data bank (Pdb) accession codes are provided. IAA, indole‐3‐acetic acid; IPA, indole‐3‐pyruvic acid.

One of the bacterial strains used by Conway et al. (2022) in their auxin metabolic signal interference assays was *Pseudomonas putida* 1290 – the first bacterium in which a cluster for IAA degradation was described and characterized (Leveau & Gerards, 2008). This IAA degrading strain is also the first bacterium for which chemoattraction to IAA has been observed, as reported recently in *Environmental Microbiology* (Rico‐Jiménez et al., 2022). This chemotactic behaviour was found to be independent of auxin metabolism and subsequent research resulted in the identification of the first chemoreceptor, named PcpI, which mediates chemotaxis to IAA. The magnitude of the tactic response towards IAA correlated with the expression of *pcpI* and the heterologous expression of the PcpI chemoreceptor in environmental and human‐associated bacteria conferred these microorganisms the ability to migrate chemotactically to IAA (Rico‐Jiménez et al., 2022). One of the main functions of chemotaxis is to provide access to nutrients and favourable niches for growth (Colin et al., 2021). Although PcpI was not found to play a relevant role in host establishment, root colonization is a multifactorial phenomenon in which multiple chemoreceptors can participate in a coordinated manner (Feng et al., 2019). Consistent with this data, a large number of diverse chemoreceptors with unknown functions are encoded in the genome of *P. putida* 1290 (Rico‐Jiménez et al., 2022). Advancing our understanding of the ligands that are recognized by bacterial chemoreceptors may provide the basis for engineering plant growth‐promoting bacteria with improved host colonizing capacity.

Antagonism of plant pathogenic microorganisms is one of the strategies by which beneficial phytobacteria promote plant growth (Roca & Matilla, 2023). The production of the antibiotic andrimid, active against important bacterial phytopathogens like *Xanthomonas campestris* and *Dickeya solani*, was shown to be regulated in a biocontrol rhizosphere isolate by the pathway‐specific transcriptional regulator AdmX. This regulatory protein was found to bind IAA and indole‐3‐pyruvic acid (IPA). However, only IAA binding to AdmX, but not IPA, modulated antibiotic production (Matilla et al., 2018). This experimental evidence has motivated the use of AdmX as a model to examine the role of auxins as agonists and antagonists (Gavira et al., 2023). To determine the molecular detail of this phenomenon, the 3D structure of the sensor domain of AdmX was solved in the presence of IAA and IPA (Figure 1). It was found that both auxins compete for AdmX binding and that IPA binding resulted in increased compactness of the AdmX structure. Several structural differences were found between the structures of the sensor domain of AdmX with bound IAA or IPA, and molecular dynamics simulations revealed important differences in the binding mode of both auxins to AdmX (Gavira et al., 2023). Key residues for IAA and IPA binding were determined, allowing the authors to identify an auxin recognition motif. The investigation of the evolutionary history of this sensor domain supports that a plant‐bacteria co‐evolutionary process has driven the emergence of this auxin receptor protein (Gavira et al., 2023). Parallels arise to recent in vitro evolutionary experiments showing an increase in the affinity and specificity of auxin recognition by another bacterial response regulator, TrpR (Herud‐Sikimić et al., 2021).

The identification of antagonists and anti‐virulence agents that target key signal transduction systems for bacterial virulence is emerging as a promising alternative approach to antibiotics (Chadha et al., 2022; Krell & Matilla, 2022) – an aspect of enormous importance in the current global context of the increasing antibiotic resistance in plant, animal and human pathogenic (micro)organisms (Darby et al., 2022; Sundin & Wang, 2018). Given the role of indole as modulator of quorum sensing and virulence factor production in pathogenic bacteria (Lee et al., 2015; Song & Wood, 2022), indole‐mediated signalling has been proposed as a potential target for anti‐virulence therapy approaches (Lee et al., 2015). Indeed, indole was shown to reduce the virulence caused by pathogenic *Vibrio* species that are of global relevance in aquaculture (Yang et al., 2017; Zhang et al., 2022b). However, given the toxicity of indole to animals at the concentrations necessary to obtain protection against *Vibrio* pathogens, there is a need to search for indole‐related compounds with reduced toxicity. To this end, a recent study in *Microbial Biotechnology* has examined the effect of multiple indole analogues on virulence, biofilm formation and motility of *Vibrio campbellii* (Zhang et al., 2022a). The authors found that different auxins, including IAA and IPA, negatively impact on the motility and biofilm‐forming capacity of *V. campbellii*. Remarkably, several indole analogues were shown to protect brine shrimp larvae against *V. campbellii* infection in a process that did not inhibit pathogen growth ‐ in accordance with the anti‐virulence therapy concept. Among these molecules, the natural auxin indole‐3‐acetonitrile was found to be the most bioactive molecule against *V. campbellii*, being extremely efficient in protecting shrimp larvae at concentrations as low as 10 μM (Zhang et al., 2022a). Taken together, these results lay the groundwork for the design of novel indole analogues with improved biological properties.

## CONCLUDING REMARKS AND FUTURE PERSPECTIVES

The function of auxins as plant hormones was first described in the 1920s. This initial knowledge has now led to their consideration as key inter‐ and intra‐kingdom signalling molecules in life (Duca & Glick, 2020; Kunkel & Johnson, 2021; Lin et al., 2022; Nicastro et al., 2021). However, regardless of the extensive knowledge that exists in plants about the mechanisms by which auxins carry out their activities, we are only beginning to understand the functions and the signalling mechanisms by which auxins carry out their biological activities in alternative (micro)organisms. Nonetheless, recent research outlined here has identified novel auxin‐sensing proteins (Figure 1) and laid the foundations for the biotechnological and sanitary applicability of auxins, which can act either as agonists or antagonists of specific microbial functions. Among the applications derived from this knowledge are: (i) the modulation of the catabolic potential of microorganisms (Conway et al., 2022; Laird et al., 2020); (ii) the alteration of the production of antibiotics of clinical and agricultural interest (Gavira et al., 2023; Matilla et al., 2018); (iii) their use as virulence inhibitors in anti‐infective therapy strategies (Zhang et al., 2022a); (iv) the development of new biosensors (Herud‐Sikimić et al., 2021) and (v) the engineering of plant microbiomes as a strategy to promote plant growth (Conway et al., 2022). In addition, microbial auxins were shown to promote microalgae growth and, consequently, the yield of high‐value products of clinical and industrial interest by these increasingly important living bioreactors (Lin et al., 2022). In this regard, an auxin‐inducible system from plants has been used to engineer yeasts with enhanced terpene production capacities (Hayat et al., 2021).

## AUTHOR CONTRIBUTIONS

**Tino Krell:** Funding acquisition (equal); project administration (equal); writing – review and editing (equal). **José A. Gavira:** Funding acquisition (equal); project administration (equal); writing – review and editing (equal). **Amalia Roca:** Funding acquisition (equal); project administration (equal); writing – review and editing (equal). **Miguel A. Matilla:** Conceptualization (equal); funding acquisition (equal); project administration (equal); writing – original draft (lead); writing – review and editing (equal).

## CONFLICT OF INTEREST STATEMENT

The authors declare that there is no conflict of interest.
